# Urine lipoarabinomannan point-of-care testing in patients affected by pulmonary nontuberculous mycobacteria – experiences from the Danish Cystic Fibrosis cohort study

**DOI:** 10.1186/s12879-014-0655-4

**Published:** 2014-12-04

**Authors:** Tavs Qvist, Isik S Johansen, Tania Pressler, Niels Høiby, Aase B Andersen, Terese L Katzenstein, Stephanie Bjerrum

**Affiliations:** Department of Infectious Diseases, Copenhagen University Hospital Rigshospitalet, Copenhagen, Denmark; Department of Infectious Diseases, Odense University Hospital, Odense, Denmark; Department of Clinical Microbiology, Copenhagen University Hospital Rigshospitalet, Copenhagen, Denmark

**Keywords:** Lipoarabinomannan, LAM, NTM, Nontuberculous, Abscessus, Avium, CF

## Abstract

**Background:**

The urine lipoarabinomannan (LAM) strip test has been suggested as a new point-of-care test for active tuberculosis (TB) among human immunodeficiency virus (HIV) infected individuals. It has been questioned if infections with nontuberculous mycobacteria (NTM) affect assay specificity. We set forth to investigate if the test detects LAM in urine from a Danish cystic fibrosis (CF) population characterized by a high NTM prevalence and negligible TB exposure.

**Method:**

Patients followed at the Copenhagen CF Center were comprehensively screened for pulmonary NTM infection between May 2012 and December 2013. Urine samples were tested for LAM using the 2013 Determine™ TB LAM Ag strip test.

**Results:**

Three-hundred and six patients had a total of 3,322 respiratory samples cultured for NTM and 198 had urine collected (65%). A total of 23/198 (12%) had active pulmonary NTM infection. None had active TB. The TB-LAM test had an overall positive rate of 2.5% applying a grade 2 cut-point as positivity threshold, increasing to 10.6% (21/198) if a grade 1 cut-point was applied. Among patients with NTM infection 2/23 (8.7%) had a positive LAM test result at the grade 2 cut-point and 9/23 (39.1%) at the grade 1 cut -point. Test specificity for NTM diagnosis was 98.3% and 93.1 for grade 2 and 1 cut-point respectively.

**Conclusions:**

This is the first study to assess urine LAM detection in patients with confirmed NTM infection. The study demonstrated low cross-reactivity due to NTM infection when using the recommended grade 2 cut-point as positivity threshold. This is reassuring in regards to interpretation of the LAM test for TB diagnosis in a TB prevalent setting. The test was not found suitable for NTM detection among patients with CF.

**Electronic supplementary material:**

The online version of this article (doi:10.1186/s12879-014-0655-4) contains supplementary material, which is available to authorized users.

## Background

The prevalence of nontuberculous mycobacteria (NTM) infection among patients with cystic fibrosis (CF) in Copenhagen rose from 7.4% in 2011 to 13.0% in 2014 [1],[2]. While NTM were previously thought to be transient colonizers of the CF lung, the two types of NTM seen in Danish CF patients; *Mycobacterium abscessus* complex (MABSC) and *Mycobacterium avium* complex (MAC) are now recognized as pathogens that can seriously affect morbidity and mortality [3]. The diagnosis of clinical significant pulmonary infection caused by NTM is a challenge and relies on culture of respiratory secretions and clinical criteria set forth by the American Thoracic Society (ATS) [4].

Lipoarabinomannan (LAM) is found in the outer cell wall of mycobacterial species, and has gained attention as a possible TB diagnostic target [5]. Antigen detection assays based on LAM are available both in the format of enzyme-linked immunosorbent assays (ELISAs) and as a point-of-care test. During mycobacterial infection, LAM is released from metabolically active or degrading mycobacterial cells into the blood stream with subsequent filtration by the kidneys, passing into urine [6],[7]. Clinical evaluation of the urine LAM test has consistently shown promising results for diagnosing TB among people living with HIV in resource constrained settings, although test sensitivity and specificity varies considerably among studies and in relation to the degree of immunodeficiency [8]-[12]. Two reviews of LAM tests have argued that false positive results could be caused by colonization of NTM and point to this as a key unresolved issue [6],[7]. No studies have looked specifically at excretion of LAM from NTM patients or addressed the extent to which NTM infection affects LAM test performance. We set forth to investigate LAM test performance in the Danish CF population characterized by a high NTM prevalence and no reported TB cases.

## Methods

### Patients and setting

The Copenhagen CF Center cares for 100 children and 216 adults with cystic fibrosis, accounting for 70% of the Danish CF population [13]. No TB case has ever been reported in this population despite extensive monitoring since the mid 1980ies. Patients are seen for clinical exams in an outpatient clinic every 4 weeks their entire lives. All patients have either chest radiograph or high-resolution computer tomography (HRCT) scans performed once a year. Since 2011, all CF patients are screened systematically once a year for mycobacteria with acid-fast microscopy and mycobacterial culture performed at the National Reference Laboratory of Mycobacteriology at Statens Serum Institute. Additionally from 2012, supplementary *in-house* mycobacterial culture is performed on all sputum samples, collected every four weeks. The CF Center used ATS criteria to classify NTM patients [4]. The criteria for NTM infection are: Pulmonary symptoms, nodular or cavitatory opacities on chest radiograph, or a HRCT scan that shows multifocal bronchiectasis with multiple small nodules after appropriate exclusion of other diagnoses. In addition, positive culture results from at least 2 separate sputum samples or positive culture results from at least 1 bronchial lavage. Subjects were included prospectively; all patients attending the CF center were eligible for inclusion if they had both respiratory samples and urine available for testing.

### Respiratory samples and isolates

All respiratory samples were prospectively collected and consisted of expectorated sputa, laryngeal aspirates or bronchial lavage fluids. Samples were pre-treated with NaOH-N-acetyl cysteine to prevent bacterial overgrowth and inoculation was done on solid (Löwenstein-Jensen slants, SSI Diagnostica, Hilleroed, Denmark) and liquid culture media (MGIT 960, Becton Dickinson Microbiology Systems, Sparks, MD, USA) and incubated at 35°C to 37°C for 8 weeks. Locally, *Burkholderia cepacia* selective agar BCSA (Biomérieux) was used as growth medium for 14 days at 37 degrees Celsius with CO_2_. In case of a recent history of *Aspergillus* colonization, samples were pre-treated with 100 μL of amphotericin B. In case of growth of acid-fast bacilli, colonies were subcultured on 10% Blood Agar Base for identified by MALDI-TOF and 16S rRNA sequencing locally. Positive cultures were further identified to the species-level by a PCR based technique targeting the 23S rRNA gene followed by reverse hybridization and line probe assay (Hain Lifescience, Germany) and/or growth ability on Löwenstein-Jensen slants with 5% NaCl.

### LAM testing

For the purpose of the study, urine was collected in sterile containers during outpatient visits and frozen within 60 minutes to -20°C and within 24 hours transferred to a -80°C freezer. Frozen urine samples were thawed to ambient temperature before applying The Determine™ TB LAM Ag test (Lot # 130103). Each sample was placed in a vortex briefly to ensure a homogenous mixture, from which 60 μL was aspirated and placed on the bottom of the sample pad. Between 25 and 35 minutes later the test was read and scored by two individuals (readers), who were blinded to their counterpart’s observations and to patient identity as well as the outcome of sputum analysis, through the use of anonymous study ID’s.

### Interpretation of the LAM test

LAM test were performed and graded by study staff with previous experience in LAM testing and interpretation. Both readers visually inspected the strip and graded the intensity of any visualized test band by comparing test band color intensity to the color intensity of the series of bands on a manufacturer-supplied paper reference card. For this study, we made use of the original 2013 reference card provided with the commercial assay of the Determine TB-LAM test. The reference scale consists of 5 color intensity grades. The test band was graded as zero if no visual band appeared and graded 1 through 5 for a visualized band of equal intensity as those on the reference card. The mean values of reader 1 and 2’s recorded test band grade were used for data analysis except for analysis of inter-reader agreement. While we report results for both grade 2 and 1 cut-point, defined as a band of equal or greater intensity to the grade 2 and 1 cut-point respectively, we acknowledge that a positive result is defined as a band of equal or greater intensity to the grade 2 cut-point in a recent consensus paper on LAM test [14].

### Clinical data collection

Clinical data including renal clearance measurements and MBL genotypes were extracted from patient files and the Danish CF registry. Data on NTM specific IgG antibody level, were captured from an ongoing study previously reported [15].

### Statistical methods

We compared urine LAM results to patients with culture positive pulmonary NTM infection and patients with no evidence of NTM infection. Sensitivities, specificities and likelihood ratios (positive and negative) were calculated with 95% CIs. Receiver operator characteristic (ROC) analysis was performed to evaluate LAM test sensitivity and specificity based on the two different band intensity thresholds for LAM positivity. Inter-reader agreement was determined using Cohen's kappa coefficient. Baseline data were calculated as medians and interquartile ranges (IQR) for continuous variables, and percentages for categorical variables. Group comparisons were made using analysis of variance or Kruskal Wallis non-parametric tests. SPSS version 19.0 (SPSS Inc, Chicago, IL) was used for data analysis.

### Ethical considerations

All participants received written and oral information about the purpose of the study and gave oral consent before clinical samples were taken. For children below 18 years informed consent was obtained from their parent or legal guardian. Exemption from the requirement of written consent was given by the Committee on Health Research Ethics in the Capital Region of Denmark, who approved the study (H-3-2012-098). Data handling was approved by the Danish Data Protection Agency (2007-58-0015).

## Results

**Figure 1:**
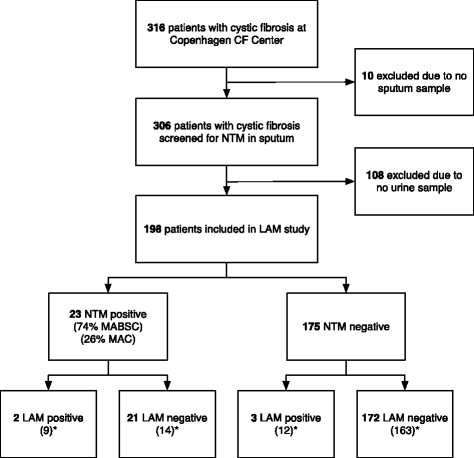
**Flow chart of cystic fibrosis patients included in the LAM study.** LAM = Lipoarabinomannan, NTM = nontuberculous mycobacteria, MABSC = *Mycobacterium abscessus* complex, MAC = *Mycobacterium avium* complex. (n) *Number of LAM positive patients if the grade 1 cut-point was used.

A total of 3,322 NTM cultures were performed on samples from 306 subjects, accounting for 97% of all patients at the Copenhagen CF Center, between May 2012 and December 2013. Of the 306 NTM screened CF patients, 198 (65%) had a urine sample collected and were included in the study. The average number of NTM cultures per patient was 9.9 (range: 1-23). A study outline including overall results is shown in Figure 1.

### Characteristics of CF population

Among the 198 patients included in the study, 23 patients (12%) had at least one positive NTM culture within 12 months prior to LAM testing. Of these, 19 (83%) fulfilled ATS criteria for NTM disease and had been NTM infected a median of 3 years (IQR: 2 – 4 years) with a total of 276 positive NTM cultures combined. Fifteen patients (65%) received NTM treatment at the time of LAM testing. MABSC regiments typically consisted of moxifloxacin combined with a macrolide and either inhaled or intravenous amikacin, and for MAC: Myambutol, clarithromycin and rifampicin. No CF patient had positive cultures for *Mycobacterium tuberculosis* complex.

### Distribution of determine TB-LAM test results in the CF population

All LAM test had a valid test result with a control bar appearing. The distribution of LAM test grading was: No band (grade 0), 177/198 (89.4%); grade 1, 18/198 (8.1%); grade 2, 1/198 (0.5%); grade 3, 2/198 (1.0%); grade 4, 2/198 (1.0%); grade 5, 0/198 (0%). As such, a total of 5 CF patients (2.5%) were LAM test positive when using a grade 2 cut-point. When lowering the positivity threshold to cut-point 1 we identified 21 LAM positive cases, increasing the positive rate to 10.6%. As there were no past or present cases of TB, all LAM positive results can be considered false positive in evaluation of the test performance for TB diagnosis.

**Figure 2:**
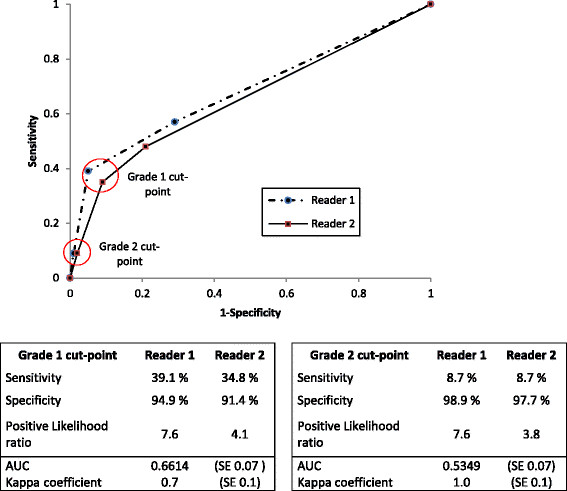
**Receiver operator characteristic (ROC) curve for urine LAM test graded by two independent readers.** Sensitivity, specificity, positive likelihood ratios, AUC and Kappa coefficient for grade 1 and grade 2 cut-point. LAM = Lipoarabinomannan.

Inter-rater agreement between reader 1 and 2 was 1.0 (SE 0.07) and 0.7 (SE 0.07) for grade 2 and grade 1 cut-point, respectively, calculated with kappa coefficient (Figure 2).

### TB LAM operating characteristics and NTM cross-reactivity

Receiver operating characteristic curve analysis showed that a positivity threshold of grade 2 cut-point minimized sensitivity, increased specificity and had better inter-rater agreement (Figure 2). Among patients with NTM infection 2/23 (8.7%, 95% CI: 1.3 – 28.1) had a positive LAM test result at the grade 2 cut-point and 9/23 (39.1%, 95% CI: 19.7 – 61.5) at the grade 1 cut -point. Test specificity for NTM diagnosis was 98.3% (95% CI: 95.1 - 99.6) and 93.1% (95% CI: 88.3 – 96.4) for grade 2 and 1 cut-point respectively. The positive likelihood ratio (LR+) was 5.1 (95% CI: 0.9 – 28.8) for grade 2 cut-point and statistically insignificant. The LR+ for grade 1 cut-point was 5.7 (95% CI: 2.7-12-0). TB-LAM test properties for NTM diagnosis (sensitivity, specificity and likelihood ratios) are shown in Table 1.

**Table 1:** **Performance of urine LAM test to detect NTM in 198 cystic fibrosis patients in Denmark**

			Sensitivity		Specificity		PLR	NLR
			N	% (95% CI)	N	% (95% CI)	(95% CI)	(95% CI)
**Grade 1 cut-point**			9/23	39.1 (19.7-61.5)	163/175	93.1 (88.3-96.4)	5.7 (2.7-12.0)	0.7 (0.5-0.9)
**Grade 2 cut-point**			2/23	8.7 (1.3-28.1)	172/175	98.3 (95.1-99.6)	5.1 (0.9-28.8)	0.9 (0.8-1.1)

PLR = Positive Likelihood Ratio, NLR = Negative Likelihood Ratio.

### Factors associated with a positive LAM test among patients with verified NTM infection

Two NTM species were observed among the 23 culture positive patients, MABSC (74%) and MAC (26%). When using the cut-point 2 only two patients with NTM were LAM positive. These two were female, 20 and 12 years old, one was newly infected with MABSC and not yet in NTM treatment at the time of urine sampling; the other had received extensive NTM treatment for an unusually aggressive MABSC infection with consistently high bacterial loads in respiratory samples. Both had significant clinical and radiological deterioration as result of their infection. When stratifying NTM patients according to the cut-point 1 for LAM positivity, nine patients were positive and 14 were negative (Table 2). The proportion of female patients was higher among LAM positive than LAM negative NTM patients (80% vs. 30%, *p* = 0.04). Likewise the proportion of patients carrying a defective MBL gene was higher among LAM positive than LAM negative patients (56% vs. 7%, *p* = 0.01) despite a similar distribution of MBL alleles when comparing the 23 NTM patients to the background CF population (data not shown). No other statistically significant differences between LAM positive and LAM negative NTM patients were observed, including rate of chronic renal disease (*p* = 0.70), treatment rate (*p* = 0.58) or NTM specific IgG antibody levels (*p* = 0.08, data not shown), measured by a previously described ELISA method [15].

**Table 2:** **Characteristics of NTM culture positive patients stratified by LAM result at grade 1 cut-point**

Characteristic at time of first positive NTM culture	LAM positive ( *n* = 9)	LAM negative ( *n* = 14)	*p* -value
Median age (IQR), y	19.7 (13.7 – 25.6)	16.1 (11.9 – 24.0)	0.41
Female, %	88.9	28.6	0.01
Homozygote for Delta 508, %	78.8	85.7	0.62
Median FEV1% of pred. (IQR)	80.0 (70.3 – 84.8)	70.0 (66.3 – 83.5)	0.59
Diabetes mellitus, %	11.1	28.6	0.32
Structurally defective MBL allele*, %	55.6	7.1	0.01
Other chronic infection, %	77.8	50.0	0.18
Received NTM treatment, %	55.6	57.1	0.94
Number of positive NTM cultures	11.0 (3.5 – 19.5)	6.0 (1.8 – 14.5)	0.68
MABSC, %	88.9	64.3	0.19
Fulfilled ATS criteria for NTM, %	88.9	78.6	0.52
Median time positive for NTM (IQR), y	2.5 (1.0 – 4.0)	3.0 (1.0 – 5.0)	1.00
Chronic renal disease**, %	0	14.3	0.24

MABSC = *M. abscessus* complex, MAC = *M. avium* complex, NTM = nontuberculous mycobacteria, IQR = Interquartile range, FEV1% = forced expiratory volume in 1 second as percent of predicted for age, height and sex, ATS = American Thoracic Society.

*XA/B (n = 3) or YA/B (n = 3) genotype.

**Defined as renal-clearance below 107 ml/min for adult men and 87 ml/min for adult women or in case of missing renal-clearance measurement, a mean serum creatinine at time of LAM testing over 105 or 90 μmol/L for adult men and women respectively or > 35, 40, 50, 55, 65 or 70 μmol/L for age groups 3-5 y, 6-7 y, 8-9 y, 10-11 y, 12-13 y and 14-15 y respectively.

## Discussion

This is the first study to investigate urine LAM strip test performance in Danish CF patients infected with pulmonary NTM. At the grade 2 cut-point (“2013 reference card”) the LAM test had a sensitivity of 8.7% and specificity of 98.3% to detect NTM infection, which is below the acceptable performance level for NTM diagnosis among CF patients. However, the primary clinical utility of LAM point-of-care testing is not to detect NTM infection, but TB in HIV positive patients from high TB burden settings [6],[7],[16]. In this context pulmonary NTM infection has previously been suspected of contributing to false positive LAM tests results, and the potential impact of NTM infections on LAM assay specificity questioned [7]. Our results can inform this debate – although carried out in a very different population. It is important to note that the TB-LAM manufacturers changed the reference card in 2014 omitting the weakest color grade (grade 1 cut-point) such that grade 1 cut-point for the new 2014 reference card corresponds to the grade 2 cut-point at the original 2013 reference card. The modification was based on previous LAM test studies [12],[17] and the recommendations in a consensus paper by Lawn et al. [14]. To increase the evidence base we have reported results for both grade 1 and grade 2 cut-points. At the recommended grade 2 cut-point a low sensitivity (8.7%) and insignificant positive likelihood ratio demonstrate a limited rate of LAM test cross-reactivity of pulmonary NTM infection. At the grade 1 cut-point we found a higher sensitivity of 39.1% and a positive likelihood ratio that demonstrated statistically convincing evidence of cross-reactivity of NTM infection. The results implies that NTM infection is likely to be one of the factors contributing to the variation in LAM test specificity as suggested in a review of the LAM test to diagnose TB in HIV infected individuals [7]. Furthermore, LAM test cross-reactivity of NTM infection could partially explain the increase in specificity reported when switching from a grade 1 cut-point to grade 2 cut-point in recent evaluations of the LAM test for TB diagnosis [12],[17]. LAM is a component of both the TB and NTM cell wall and structural differences in LAM across mycobacterial species are well described [18]. This diversity of LAM molecules makes it likely, that different mycobacteria have different thresholds for test positivity in a LAM assay. Our study provides evidence for excretion of LAM from pulmonary NTM patients, although this excretion is apparently at lower concentrations, than that seen for active TB. The reason for this might be a lower bacterial burden in patients with NTM infection or diversity of LAM molecules. Indeed an *in-vitro* analysis of a LAM ELISA prototype showed that LAM could be detected from patients colonized with NTM, albeit only at significantly higher mycobacterial concentrations than TB [19].

The minimized risk of cross-reactivity from NTM infection observed in our study setting when using grade 2 cut-point (corresponding to the new grade 1 cut-point) is reassuring for the future use of LAM testing for TB diagnosis using the 2014 reference card. However, the need remains to assess the association between detection of urine LAM and NTM infection among the appropriate target population i.e. HIV positive patients from high TB burden settings. Four TB studies have reported a total of 10 cases of LAM positivity in HIV-positive patients with NTM infection instead of TB [8],[11],[20],[21], but information on NTM species was not reported. One of the papers, a clinical study on 291 patients from Tanzania, found LAM reactivity in four TB suspected patients, who were later found culture positive for NTM. The authors regarded the finding as background false-positivity [21], but our findings challenge this conclusion and instead suggest that genuine cross-reactivity to NTM was a likely explanation.

The impact of NTM infection on LAM test accuracy also depends on NTM prevalence among HIV-positive patients. The burden of TB is the leading cause of morbidity and mortality in HIV patients [22],[23]. A recent autopsy study among HIV positive individuals in South Africa found *M. tuberculosis* in 26/39 (67%) of the mycobacterial cases and comparatively 2/39 (5%) with NTM [24]. This confirms findings in a previous meta-analysis of all autopsy studies of HIV patients from Sub-Saharan Africa were TB was considered a cause of death in 32-45% of 593 autopsied adults [25]. However, there are reports that NTM disease could be underreported in some settings [26],[27] and that misclassification of NTM as TB might occur [28]-[30]. A study from Nigeria recently found a NTM prevalence of 15% among patients with presumptive TB [31]. Unlike TB, NTM are environmental pathogens with surprising disparity in incidence in different geographical regions [4]. The reasons for this heterogeneity in NTM pulmonary disease distribution is not well understood and even less is known about these factors in Sub-Saharan Africa.

The question of whether the LAM point-of-care test could be used to screen for pulmonary NTM infection in a resource constrained setting remains open. This would certainly require supplementary species discrimination, but given the manufacturers change in reference card and the low sensitivity at the grade 2 cut-point, the test has no immediate applicability for this purpose. Additionally, in a Western European setting, LAM testing for the purpose of NTM diagnosis is inferior to the readily available gold standard: mycobacterial culture, microscopy and PCR.

Why NTM patients who were LAM positive using cut-point 1 were statistically more likely to carry a MBL variant allele and were more likely to be female remains unknown. It is well described that female patients have worse clinical outcomes in CF [32], but there were no statistically significant differences in lung function, renal clearance or CF mutations in the present comparison to further explain this association. MBL insufficiency in CF patients is associated with a poorer prognosis, specifically earlier acquisition of chronic Gram-negative infection and progression to end stage lung disease [33]-[35]. Previous TB studies have suggested that carriers of MBL variant alleles might be either more [36],[37] or less [38],[39] susceptible to TB infection, but to our knowledge this has not previously been studied for NTM. The NTM patients in the current study had the same distribution of MBL alleles as the background CF population.

It has been proposed that excretion of LAM in urine may depend on the kidneys ability to retain immune-bound LAM [40]. In our study we could not confirm any link between test sensitivity and chronic renal disease as measured by renal clearance rates. Nor was there any association between NTM specific IgG levels and LAM positivity, as might be expected if immune-complexes influenced the level of antigenuria. Although not statistically significant, a higher proportion of patients who were positive using cut-point 1 had a concomitant, chronic Gram-negative pulmonary infection, most commonly *Pseudomonas aeruginosa* followed by *Stenotrophomonas maltophilia*. While *Nocardia* species have been shown to cause cross-reaction to LAM-test, this has never been shown for Gram-negative bacteria [9]. *Norcardia* are seen among CF patients, but no LAM cases in the present study had a history of *Norcadia* infection. The reasons for the background false positivity seen at both cut-points could thus not be answered.

Study limitations include that 35% of the patient population were excluded due to lack of urine. However, no selection bias could be identified as patients were consecutively enrolled during routine visits independently of clinical or microbiological characteristics. A majority of NTM patients received NTM specific treatment at the time of urine collection, which might affect LAM test performance by reducing the mycobacterial burden.

The primary strength of the study was the prospective design in a well-described homogenous population with unparalleled visit-frequency, negligible TB exposure and a mean of 10 mycobacterial cultures per included individual.

## Conclusion

This study of a comprehensively screened CF population demonstrated a low level (8.7%) of LAM excretion from patients with NTM infection when using the recommended grade 2 cut-point as positivity threshold, but high level (39.1.%) at the outdated grade 1 cut-point. This is reassuring for the intended utility of the revised “2014” LAM test to diagnose TB among patients infected with HIV in resource constrained settings, where access to mycobacterial cultures is limited. The LAM strip test was not suitable for NTM diagnostic use in cystic fibrosis patients due to superior alternatives.

## Authors’ original submitted files for images

Below are the links to the authors’ original submitted files for images. Authors’ original file for figure 1 Authors’ original file for figure 2

## Abbreviations

LAM: Lipoarabinomannan
TB: Tuberculosis
HIV: Human immune deficiency virus
CF: Cystic fibrosis
NTM: Nontuberculous mycobacteria
MABSC: *Mycobacterium abscessus* complex
MAC: *Mycobacterium avium* complex
ELISA: Enzyme-linked immunosorbent assay
BSCA: *Burkholderia cepacia* selective agar
HRCT: High resolution computer tomography
ATS: American thoracic society
IQR: Interquartile range
CI: Confidence interval
SE: Standard error
FPR: False positive rate
FEV1%: forced expiratory volume in 1 second as percent of predicted for age, height and sex
