# Supplementary figures and images for: Urine lipoarabinomannan point-of-care testing in patients affected by pulmonary nontuberculous mycobacteria – experiences from the Danish Cystic Fibrosis cohort study
Source: BMC Infect Dis. 2014 Dec 4;14:655. doi: 10.1186/s12879-014-0655-4 (PMC4260379; doi:10.1186/s12879-014-0655-4)

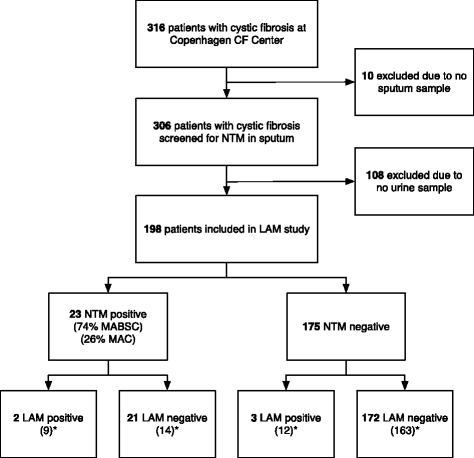

Supplement: Supplementary file 1 — Authors’ original file for figure 1 [file 12879_2014_655_MOESM1_ESM.gif]

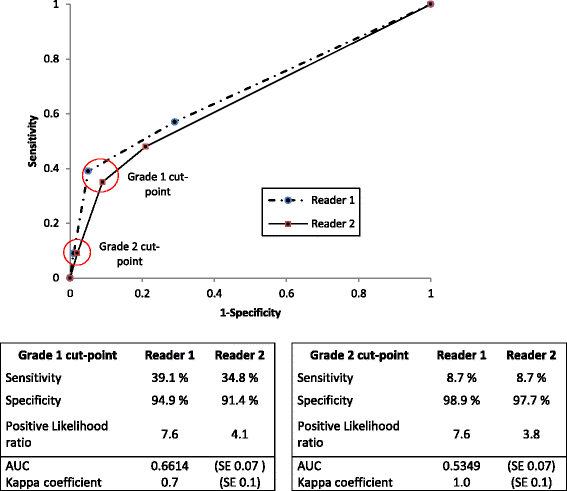

Supplement: Supplementary file 2 — Authors’ original file for figure 2 [file 12879_2014_655_MOESM2_ESM.gif]
